# Release the BEESTS: **B**ayesian **E**stimation of **E**x-Gaussian **ST**op-**S**ignal reaction time distributions

**DOI:** 10.3389/fpsyg.2013.00918

**Published:** 2013-12-10

**Authors:** Dora Matzke, Jonathon Love, Thomas V. Wiecki, Scott D. Brown, Gordon D. Logan, Eric-Jan Wagenmakers

**Affiliations:** ^1^Department of Psychological Methods, University of AmsterdamAmsterdam, Netherlands; ^2^Laboratory of Neural Computation and Cognition, Brown UniversityProvidence, RI, USA; ^3^School of Psychology, University of NewcastleCallaghan, NSW, Australia; ^4^Department of Psycholgy, Vanderbilt UniversityNashwille, TN, USA

**Keywords:** stop-signal paradigm, stop-signal RT distribution, ex-Gaussian distribution, hierarchical Bayesian modeling, statistical software

## Abstract

The stop-signal paradigm is frequently used to study response inhibition. In this paradigm, participants perform a two-choice response time (RT) task where the primary task is occasionally interrupted by a stop-signal that prompts participants to withhold their response. The primary goal is to estimate the latency of the unobservable stop response (stop signal reaction time or SSRT). Recently, Matzke et al. (2013) have developed a Bayesian parametric approach (BPA) that allows for the estimation of the entire distribution of SSRTs. The BPA assumes that SSRTs are ex-Gaussian distributed and uses Markov chain Monte Carlo sampling to estimate the parameters of the SSRT distribution. Here we present an efficient and user-friendly software implementation of the BPA—BEESTS—that can be applied to individual as well as hierarchical stop-signal data. BEESTS comes with an easy-to-use graphical user interface and provides users with summary statistics of the posterior distribution of the parameters as well various diagnostic tools to assess the quality of the parameter estimates. The software is open source and runs on Windows and OS X operating systems. In sum, BEESTS allows experimental and clinical psychologists to estimate entire distributions of SSRTs and hence facilitates the more rigorous analysis of stop-signal data.

## 1. Introduction

Response inhibition—the ability to stop an ongoing response—is frequently studied using the stop-signal paradigm. In the stop-signal paradigm (Logan and Cowan, 1984; Lappin and Eriksen, 1966), participants perform a two-choice visual response time (RT) task, such as responding to the color or the shape of the stimuli. This primary task is occasionally interrupted by a stop-signal that instructs participants not to respond on that trial. The goal is to estimate the latency of the unobservable stop response (stop-signal RT; SSRT).

Based on the independent horse-race model (Logan, 1981; Logan and Cowan, 1984), various methods are available to estimate SSRTs (e.g., Logan, 1994; Verbruggen and Logan, 2009; Verbruggen et al., 2009). Over the past decades, the horse-race model has been extensively used to estimate stopping latencies and compare the efficiency of response inhibition between different age groups (e.g., Schachar and Logan, 1990; Kramer et al., 1994; Ridderinkhof et al., 1999; Williams et al., 1999) and clinical populations (Schachar and Logan, 1990; Oosterlaan et al., 1998; Schachar et al., 2000). Unfortunately, most standard methods to estimate SSRTs only provide a summary measure of the latency of the stop process, such as the mean or the median SSRT.

Several researchers have argued, however, that the adequate analysis of RT data should not only focus on mean RT, but should consider the shape of the entire RT distribution (e.g., Heathcote et al., 1991; Matzke and Wagenmakers, 2009). The shape of SSRT distributions may, for example, differ between different clinical populations, without necessary differences in mean SSRT. Ignoring the shape of SSRT distributions may thus lead to incorrect conclusions about differences in response inhibition between groups.

To allow for a more thorough analysis of stop-signal data, Matzke et al. (2013) have recently developed a Bayesian parametric approach (BPA) that enables researchers to estimate the entire distribution of SSRTs (see Logan et al., in press, for an alternative approach). The BPA assumes that SSRTs follow an ex-Gaussian distribution and uses Bayesian parameter estimation to obtain posterior distributions for the model parameters. The BPA enables researchers to compare and evaluate differences in the ex-Gaussian stop parameters between experimental and clinical groups. By doing so, the BPA has the potential to facilitate the interpretation of stop-signal data and contribute to new insights on the nature of response inhibition.

Parameter estimation in the BPA currently relies on the popular Bayesian statistical package WinBUGS (Bayesian inference Using Gibbs Sampling for Windows; Lunn et al., 2012). The practical usefulness of the BPA is, however, severely limited by the disadvantages of the present implementation. The WinBUGS routine is extremely time consuming and rather user-unfriendly. For instance, WinBUGS requires several hours to produce reliable parameter estimates for a single participant and it requires several days to fit a hierarchical data set. It is therefore all but impossible for experimental and clinical psychologists to take advantage of the theoretical progress offered by the BPA.

In order to overcome this obstacle and promote the broader application of the Bayesian analysis of stop-signal data, we introduce a relatively fast, user-friendly software that allows for the estimation of entire SSRT distributions. BEESTS (**B**ayesian **E**x-Gaussian **E**stimation of **ST**op-**S**ignal RT distributions) can be applied to individual and hierarchical stop-signal data and comes with an easy-to-use graphical user interface. BEESTS provides users with summary statistics of the posterior distribution of the parameters as well as various diagnostic tools to assess the quality of the parameter estimates.

The outline of the paper is as follows. First, we describe the BPA in more detail. Second, we introduce BEESTS, present the installation instructions, and describe the various analysis and output options provided by the software. Third, we illustrate the use of BEESTS with experimental stop-signal data. The last section concludes.

## 2. The bayesian parametric approach

### 2.1. Rationale and assumptions

**Figure 1:**
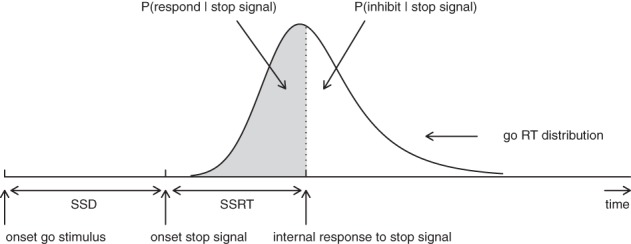
**Graphical representation of the independent horse-race model**. The success of response inhibition is determined by the relative finishing times of the go and the stop process. Primary task “go” RTs that are longer than SSD + SSRT are successfully inhibited (i.e., white area); go RTs that are shorter than SSD + SSRT escape inhibition and result in signal-respond RTs (i.e., gray area; see also Matzke et al., 2013). Constant SSRT is assumed.

According to the standard horse-race model (Logan, 1981; Logan and Cowan, 1984), performance in the stop-signal paradigm can be conceptualized as a horse-race between two independent processes that compete against each other: a go-process that is initiated by the primary task “go” stimulus and a stop-process that is generated by the stop-signal. As shown in Figure 1, if the go-process finishes before the stop-process, the primary response is executed; if the stop-process finishes before the go-process, the primary response is inhibited. The shorter the time interval between the onset of the go-stimulus and the onset of the stop-signal (i.e., stop-signal delay; SSD), the more likely participants are to inhibit their response on the primary task (see also Matzke et al., 2013).

**Figure 2:**
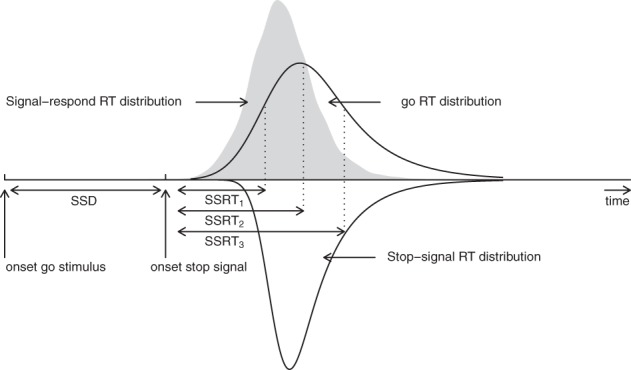
**Assumptions of the Bayesian parametric approach**. The BPA treats the distribution of signal-respond RTs (i.e., gray area) as a go RT distribution that is censored by the SSRT distribution. The censoring point can take on a different value on each stop-signal trial (e.g., SSD + SSRT_1_, SSD + SSRT_2_, and SSD + SSRT_3_). If the go RT on a given trial is longer than SSD + SSRT, the go RT is successfully inhibited. In contrast, if the go RT on a given trial is shorter than SSD + SSRT, the go RT cannot be inhibited and results in a signal-respond RT. See Matzke et al. (2013) for details.

The BPA is based on the rationale of the standard horse-race model, but it assumes that primary task “go” RTs and SSRTs are both independent random variables (i.e., complete horse-race model). As shown in Figure 2, the BPA assumes that the distribution of RTs that escape inhibition (i.e., signal-respond RTs) can be viewed as a censored go RT distribution. The censoring point is assumed to be drawn from the SSRT distribution and can take on a different value on each stop-signal trial (e.g., SSD + SSRT_1_, SSD + SSRT_2_, and SSD + SSRT_3_). The estimation of the SSRT distribution therefore involves simultaneously estimating the parameters of the go RT distribution and its censoring distribution (see also Matzke et al., 2013).

**Figure 3:**
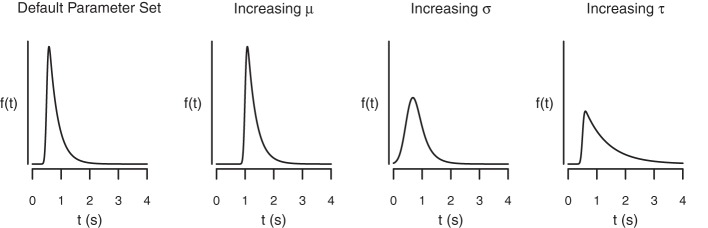
**The shape of the ex-Gaussian distribution as a function of the μ, σ, and τ parameters**. The distributions were generated with the following parameter sets: μ = 0.5, σ = 0.05, τ = 0.3 (Panel 1); μ = 1, σ = 0.05, τ = 0.3 (Panel 2); μ = 0.5, σ = 0.2, τ = 0.3 (Panel 3); and μ = 0.5, σ = 0.05, τ = 0.8 (Panel 4).

The BPA assumes that the go RTs and SSRTs are ex-Gaussian distributed (Ratcliff and Murdock, 1976; Ratcliff, 1978, 1993; Hockley, 1982, 1984; Heathcote et al., 1991; Matzke and Wagenmakers, 2009). The ex-Gaussian is a three-parameter distribution that is given by the convolution of a Gaussian and an exponential distribution. The μ and σ parameters are the mean and the standard deviation of the Gaussian component, respectively, and τ is the mean of the exponential component. The μ and σ parameters reflect the leading edge and mode of the distribution, whereas τ reflects the tail of the distribution. As shown in Figure 3, the ex-Gaussian is a positively skewed unimodal distribution that can excellently accommodate the shape of empirical RT data.

The probability density function of the ex-Gaussian is

(1) $$\begin{array}{l} f(t;\mu ,\sigma ,\tau )=\frac{1}{\tau }\exp\left(\frac{\mu -t}{\tau }+\frac{\sigma^{2}}{2\tau^{2}}\right)\;\times \\ \;\Phi \left(\frac{t-\mu }{\sigma }-\frac{\sigma }{\tau }\right)\text{for }\sigma >0,\tau >0, \end{array}$$

where Φ is the standard normal distribution function, given by

(2) $$\Phi \left(\frac{t-\mu }{\sigma }-\frac{\sigma }{\tau }\right)=\frac{1}{\sqrt{2\pi }}\int_{-\infty }^{\frac{t\;-\;\mu }{\sigma }\;-\;\frac{\sigma }{\tau }}\exp\left(\frac{-y^{2}}{2}\right)dy.$$

The mean and variance of the ex-Gaussian distribution equals

(3) E(t)=μ+τ

and

(4) $$\text{Var(t)}=\sigma^{2}+\tau^{2},$$

respectively. Note that the BPA does not assume that the ex-Gaussian parameters correspond to specific cognitive processes (Matzke and Wagenmakers, 2009); the ex-Gaussian distribution is used as a convenient descriptive model to summarize the distribution of go RTs and SSRTs. As an alternative, one may use, for instance, the ex-Wald distribution (Schwarz, 2001), or “shifted” RT distributions with a parameter-dependent lower bound, such as the shifted Wald, the shifted Weibull or the shifted log normal distribution [e.g., Heathcote, 2004; Heathcote et al., 2004; Rouder, 2005; Rouder et al., 2005; see also Luce (1986) for alternatives].

### 2.2. Bayesian parameter estimation and priors

As explained in Matzke et al. (2013), the BPA simultaneously estimates the μ_go_, σ_go_, and τ_go_ parameters of the go RT distribution and the μ_stop_, σ_stop_, and τ_stop_ parameters of the SSRT distribution. The BPA relies on Bayesian parameter estimation and therefore involves specifying the prior distribution of the model parameters. BEESTS uses slightly different priors than the WinBUGS implementation of the BPA. Note, however, that Bayesian parameter estimation is insensitive to the choice of the prior as long as sufficiently diagnostic data are available (e.g., Edwards et al., 1963; Gill, 2002; Lee and Wagenmakers, in press). The prior distributions of the model parameters for the BEESTS implementation are listed in the Appendix. The ability of BEESTS to recover underlying true parameter values with the present prior setting has been validated in a series of simulation studies. See the supplemental materials at http://dora.erbe-matzke.com/publications.html for a summary of the results of the parameter recoveries.

**Figure 4:**
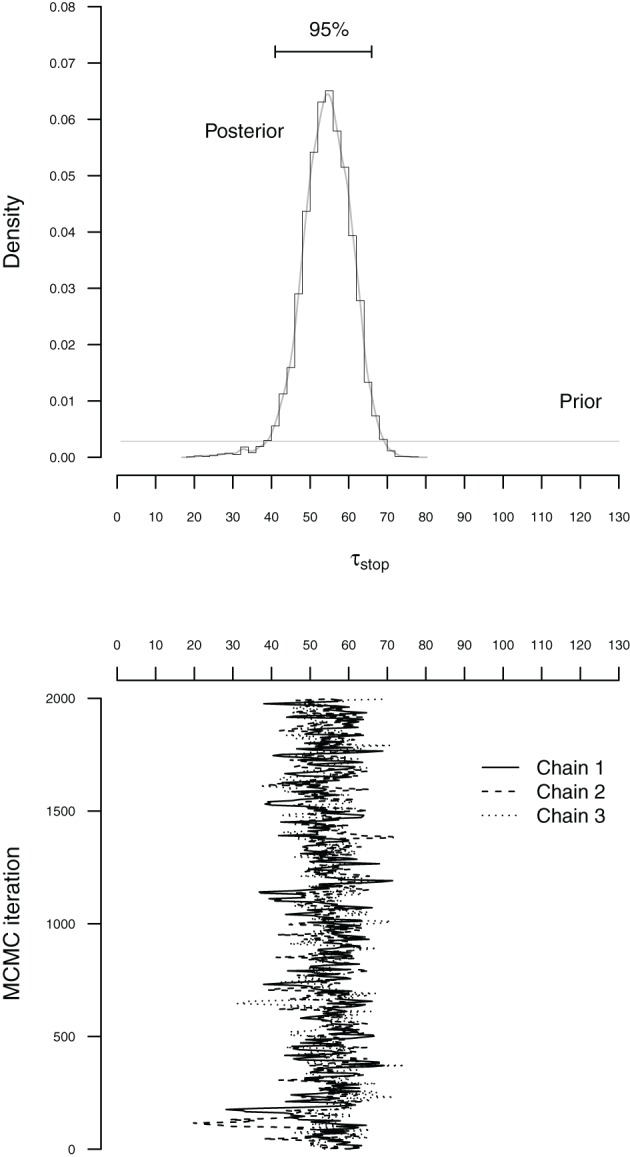
**Illustration of MCMC-based Bayesian estimation for the τ_stop_ parameter with the individual BPA**. The histogram in the top panel figure shows the posterior distribution of τ_stop_. The corresponding gray line indicates the fit of a non-parametric density estimator. The horizontal black line at the top of the top panel shows the 95% Bayesian credible interval. The horizontal gray line at the bottom of the top panel shows the prior distribution of τ_stop_. The solid, dashed and dotted lines in the bottom panel figure represent the different sequences of values (i.e., MCMC chains) sampled from the posterior distribution of τ_stop_. To create the histogram in the top panel, the sampled values were first collected across the three chains and then projected onto the *x*-axis of the top panel figure (see also Matzke et al., 2013).

The BPA relies on Markov chain Monte Carlo sampling (MCMC; Gilks et al., 1996; Gamerman and Lopes, 2006) to obtain posterior distributions for the go and stop parameters. Figure 4 illustrates the basic concepts of Bayesian parameter estimation using MCMC sampling. The bottom panel of Figure 4 shows sequences of values (i.e., MCMC chains) sampled from the posterior distribution of the τ_stop_ parameter. The accuracy of the sampling process can be increased by running multiple chains, discarding the beginning of each chain as burn-in, and by thinning the chains to decrease autocorrelation. In the present illustration, we ran three chains, each with different starting values and retained 2000 iterations per chain, resulting in a total of 6000 samples from the posterior distribution (see also Matzke et al., 2013).

The top panel of Figure 4 shows the prior and posterior distribution of the τ_stop_ parameter. The horizontal gray line at the bottom of the figure shows the prior distribution of τ_stop_. The prior is updated by the incoming data to yield the posterior distribution. The histogram and the gray density plot show the distribution of the samples drawn from the posterior distribution of τ_stop_ collapsed over the three MCMC chains. The posterior distribution quantifies the uncertainty about the estimate of τ_stop_. The central tendency of the posterior, such as the median, is often used as a point estimate of the parameter. The dispersion of the posterior, such as the standard deviation or the percentiles, quantifies the precision of the parameter estimate; the larger the dispersion, the greater the uncertainty in the estimated parameter. For example, the horizontal line at the top of Figure 4 ranges from the 2.5th to the 97.5th percentile of the posterior (i.e., 95% Bayesian credible interval), indicating that we can be 95% confident that the true value of τ_stop_ lies within this range (see also Matzke et al., 2013).

Before interpreting the parameter estimates, it is crucial to ensure that the chains have converged from their starting values to their stationary distributions. First, we verify that the posterior distributions of the model parameters are unimodal. Second, we run multiple MCMC chains and ascertain that the chains have mixed well. At convergence, the individual MCMC chains should look like “hairy caterpillars” and should be indistinguishable from one another. Lastly, we compute the $\hat{R}$ (Gelman and Rubin, 1992) convergence diagnostic measure for each model parameter. $\hat{R}$ compares the between-chain variability to the within-chain variability. As a rule of thumb, $\hat{R}$ should be lower than 1.1 if the chains have properly converged. In case of convergence problems, we recommend that users increase the number of samples, the length of the burn-in period, and the degree of thinning.

The BPA can be applied to individual as well as hierarchical stop-signal data. See Matzke et al. (2013) for the graphical representation of the individual and hierarchical BPA models. For the individual analysis, the goal is to estimate the ex-Gaussian go and stop parameters for each participant separately. In contrast, for the hierarchical analysis (e.g., Rouder et al., 2005; Gelman and Hill, 2007; Farrell and Ludwig, 2008; Matzke and Wagenmakers, 2009; Lee, 2011), the BPA assumes that the participant-level go and stop parameters are drawn from group-level distributions. The group-level distributions specify the between-subject variability of the participant-level parameters. The group-level distributions are themselves characterized by a set of group-level parameters. The goal is to simultaneously estimate the group-level parameters as well as the participant-level go and stop parameters. As explained in Matzke et al. (2013), hierarchical modeling is particularly valuable in situations with only a small number of observations per participant and moderate between-subject variability in parameter values (Gelman and Hill, 2007). In such situations, Bayesian hierarchical modeling typically yields less variable and more accurate estimates than single-level parameter estimation (Rouder et al., 2005; Farrell and Ludwig, 2008). The advantages of the hierarchical approach are less pronounced in situations with a large number of observations per participant. Similarly, in settings with only a few participants—a typical scenario in psychophysical experiments—the group-level parameters cannot be estimated precisely, a problem that diminishes the benefits of hierarchical modeling. In these cases, the individual approach may perform similarly well as the hierarchical approach.

## 3. Releasing the BEESTS

**Figure 5:**
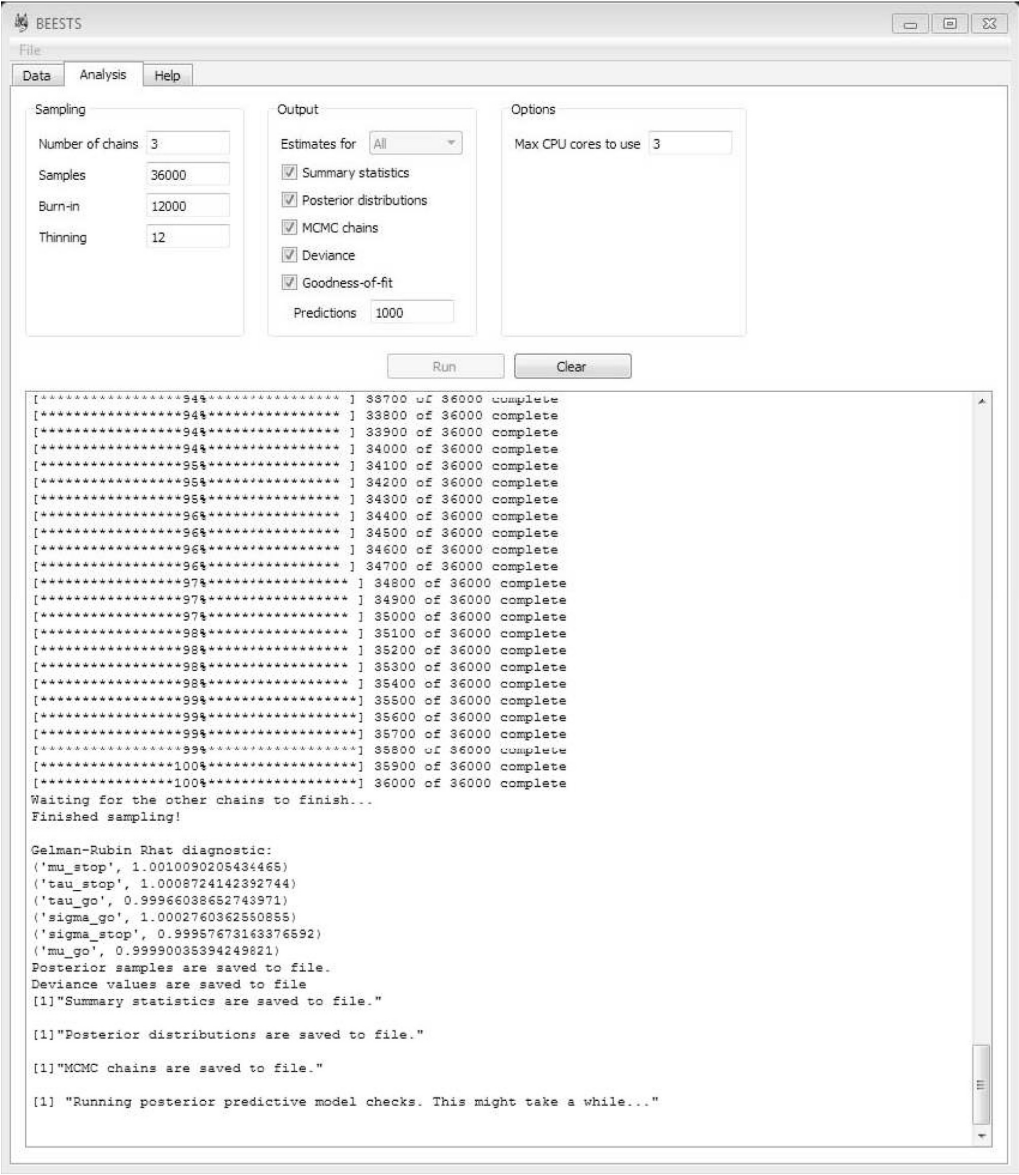
**Graphical user interface for BEESTS**. See text for details.

BEESTS is a cross-platform open-source software for the estimation of SSRT distributions with the BPA (Matzke et al., 2013). BEESTS relies on Python for parameter estimation and on R (R Core Team, 2012) for the post-processing of the posterior distribution of the model parameters. Specifically, BEESTS uses the Python-based toolboxes kabuki (Wiecki et al., 2013) and PyMC (Patil et al., 2010) to construct the model and to generate samples from the posterior distribution of the model parameters using Metropolis-within-Gibbs sampling (Tierney, 1994), respectively. For computational efficiency, the likelihood functions are coded in Cython (Behnel et al., 2011). Once the model parameters are estimated, BEESTS relies on R to compute summary statistics for the posterior distribution of the model parameters and to assess the quality of the parameter estimates. As shown in Figure 5, BEESTS is equipped with an easy-to-use graphical user interface (GUI).

## 4. Installation

BEESTS is a stand-alone and open source software released under the Affero General Public License. BEESTS runs on Windows (Windows XP and Windows 7) and OS X (Mountain Lion) operating systems. The software is freely available at http://dora.erbe-matzke.com/software.html. To install BEESTS on Windows, download BEESTS-1.2.zip and unpack the zip file at any desired location on your computer. Start the GUI by clicking on BEESTS.exe. To install BEESTS on OS X, download BEESTS-1.2.dmg, double-click the file, and install it on your computer.

## 5. Loading data

**Figure 6:**
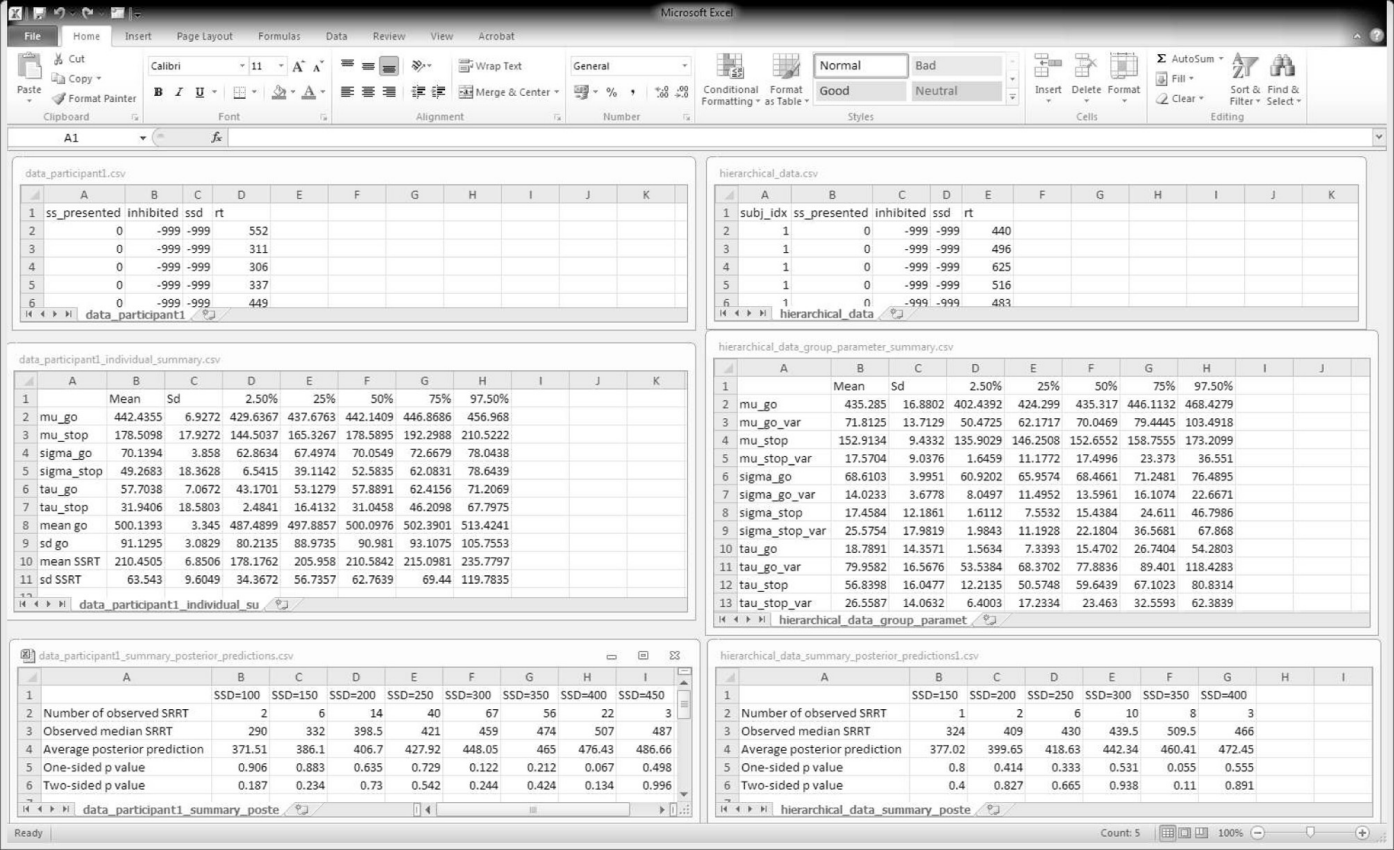
**BEESTS input and output**. The **left panels** show input and output for the individual analysis. The **right panels** show input and output for the hierarchical analysis. The **top panels** show the required data format. The **middle panels** show the output of the Summary statistic option. For the hierarchical analysis, only the group-level mean and group-level variability (i.e., standard deviation) parameters are shown. The **bottom panels** show partial output for the Goodness-of-fit option for Participant 1 in the Bissett and Logan (2011) experiment. SRRT = signal-respond RT.

The top panels of Figure 6 show the required data format for the analysis. Data files should be saved as csv (i.e., comma-separated values) files. For the individual analysis, the first row of the data file must contain the column names "ss_presented", "inhibited", "ssd", and "rt". The remaining rows contain the data for each go and stop-signal trial. For the hierarchical analysis, the first row of the data file must additionally contain the column name "subj_idx". See Table 1 for instructions on response coding and the examples folder in BEESTS for examples of the data format.

**Table 1:** **Response coding for the hierarchical BEESTS analysis**.

"subj_idx"	"ss_presented"	"inhibited"	"ssd"	"rt"
1	0	−999	−999	656
1	1	0	300	469
1	1	1	300	−999

*The* "subj_idx" *column contains the participant number. The* "ss_presented" *column contains the trial type, where go trials are coded with 0 and stop-signal trials are coded with 1. The* "inhibited" *column contains the inhibition data, where signal-respond trials are coded with 0 (i.e., unsuccessful inhibition), signal-inhibit trials are coded with 1 (i.e., successful inhibition), and go trials are coded with −999. The* "ssd" *column contains the stop-signal delay in ms., where go trials are coded with −999. The* "rt" *column contains the go RT for go trials and the signal-respond RT for signal-respond trials in ms., where signal-inhibit trials are coded with −999.*

To load the data file, click on Open in the File menu and follow the instructions. Based on the data format, BEESTS automatically infers whether an individual or hierarchical analysis is appropriate: data files without the "subj_idx" column are analyzed with the individual BPA, whereas data files with the "subj_idx" column are analyzed with the hierarchical BPA.

## 6. Analysis

Once the data are loaded, users can specify the details of the MCMC sampling, the required output, and the preferred number of CPU cores used by BEESTS.

### 6.1. Sampling

BEESTS allows users to specify the following aspects of the sampling run. Typical values of the input arguments are shown in Figure 5.

#### 6.1.1. Number of chains

Use the Number of chains option to specify the number of MCMC chains, i.e., sequences of values sampled from the posterior distribution of the parameters. The start values are automatically set to the maximum *a posteriori* probability (MAP) estimates of the parameters.

#### 6.1.2. Samples

Use the Samples option to specify the total number of MCMC samples per chain.

#### 6.1.3. Burn-in

Use the Burn-in option to specify the number of burn-in samples to discard at the beginning of each chain.

#### 6.1.4. Thinning

Use the Thinning option to specify the degree of thinning within each chain. For instance, a thinning factor of 12 means that only every 12th MCMC sample will be retained.

### 6.2. Output

All output will be saved in the directory where the data file is located. BEESTS automatically saves the posterior samples from each chain to a separate csv file (e.g., name.datafile_parameters1.csv, name. datafile_parameters2.csv, etc.). If multiple chains are run, BEESTS automatically displays the $\hat{R}$ statistic for each model parameter (see Figure 5).

As shown in Figure 5, BEESTS allows users to request the following additional output. If Estimates for is set to All in a hierarchical analysis, BEESTS will provide the selected output options (i.e., summary statistics, density plots of the posterior distributions, and MCMC trace plots) for the group-level parameters *and* for each participant separately. If Estimates for is set to Only-group, BEESTS will provide the selected output options only for the group-level parameters.

#### 6.2.1. Summary statistics

Use the Summary statistics option to obtain a csv file with the summary statistics (i.e., mean, standard deviation, and quantiles) of the posterior distribution of the model parameters and of the corresponding mean and standard deviation of the go and SSRT distribution (see Equations 3, 4).

#### 6.2.2. Posterior distributions

Use the Posterior distributions option to obtain a pdf file with the density plots of the posterior and the prior distribution of the model parameters.

#### 6.2.3. MCMC chains

Use the MCMC chains option to obtain a pdf file with trace plots for the MCMC chains of the model parameters.

#### 6.2.4. Deviance

Use the Deviance option to obtain the deviance values from each chain in a separate csv file (e.g., name.datafile_deviance1.csv, name.datafile_deviance2.csv, etc.). The deviance values may be used to compute the Deviance Information Criterion (DIC, e.g., Spiegelhalter et al., 2002) measure of model selection.

#### 6.2.5. Goodness-of-fit

Use the Goodness-of-fit option to assess the absolute goodness-of-fit of the model using posterior predictive model checks. As explained in Matzke et al. (2013), the adequacy of the model can be assessed by generating predicted data using the posterior distributions of the parameters. If the model adequately describes the data, the predictions based on the model parameters should closely approximate the observed data. The model checks can be formalized by computing posterior predictive *p* values [e.g., Gelman et al., 1996; Gelman and Hill, 2007, but see Bayarri and Berger (1998)]. Extreme *p* values close to 0 or 1 indicate that the BPA does not describe the observed data adequately.

For each individual participant, BEESTS uses the median of the observed and predicted signal-respond RTs as test statistics. The Predictions option can be used to specify the number of predicted data sets. BEESTS then randomly samples the specified number of parameter vectors from the joint posterior of the individual go and stop parameters. Next, BEESTS generates the specified number of predicted stop-signal data sets for each SSD using the corresponding number of stop-signal trials and the chosen parameter vectors. For each SSD, BEESTS then computes the median signal-respond RT in each predicted data set. Lastly, for each SSD, BEESTS computes the one-sided posterior predictive *p* value given by the fraction of times that the predicted median signal-respond RT is greater than the observed median signal-respond RT. Corresponding two-sided *p* values can be computed as 2 × min(*p*, 1 − *p*). Note, however, that two-sided *p* values are well defined only when the test statistic has a symmetric distribution. Note also that BEESTS assesses model fit on all SSDs that contain at least one observed signal-respond RT. In order to obtain stable median signal-respond RTs, however, we advise users to interpret the results only on SSDs with a reasonable number of observed signal-respond RTs.

The output of the posterior predictive model checks consists of (1) a csv file listing for each SSD the number of observed signal-respond RTs, the observed median signal-respond RT, the average of the predicted median signal-respond RTs, and the one-sided and two-sided posterior predictive *p* value and (2) a pdf file with a graphical summary of the model checks using violin plots. Violin plots (e.g., Hintze and Nelson, 1998) combine information available from density plots with information about summary statistics in the form of box plots. Note that irrespective of the type of analysis (individual or hierarchical), the goodness-of-fit of the model is assessed on a participant level using the parameter values of the individual participants (see Figure 8).

### 6.3. Options: max CPU cores to use

Use the Max CPU cores to use option to specify the number of CPU cores to use during the sampling process. If multiple MCMC chains are requested, BEESTS can run the chains in parallel by allocating each chain to a different CPU core in order to increase speed. The default number of CPU cores used by BEESTS is the number of cores available on the computer minus one.

### 6.4. Running the analysis

Once the details of the sampling process and the required output are specified, start the analysis by clicking on Run. As shown in Figure 5, BEESTS automatically displays the progress of the sampling. If multiple MCMC chains are run in parallel, BEESTS displays the progress of only one of the MCMC chains (i.e., the main process). The analysis can be stopped by “killing” the (parallel) processes in the Task Manager. Use the Clear command to clear the working space.

## 7. Empirical data examples: individual and hierarchical analysis

In this section, we illustrate the use of BEESTS with the stop-signal data of 20 participants from the 40% stop-signal condition of the first experiment reported in Bissett and Logan (2011). The data set featured a relatively large number of 720 go trials and 480 stop-signal trials per participant. See Matzke et al. (2013) for the details on the data pre-processing and the model fitting. For all of the participants, the BEESTS implementation yielded parameter estimates that are highly similar to the ones obtained from the WinBUGS routine. For a comparison of the parameter estimates from the BEESTS and the WinBUGS implementation, the reader is referred to the supplemental materials and to the empirical data examples in Matzke et al. (2013).

Due to relatively high autocorrelations between the parameters, we ran long chains, discarded the beginning of the chains as burn-in, and thinned each chain. The results reported below are based on 6000 retained samples, using Number of chains = 3, Samples = 36000, Burn-in = 12000, and Thinning = 12.

### 7.1. Individual analysis

**Figure 7:**
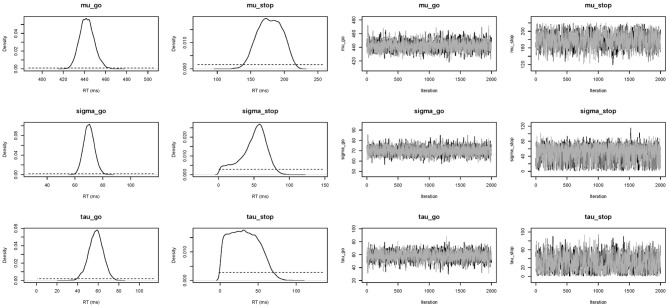
**Posterior (black solid lines) and prior distributions (black dotted lines; left panel) and MCMC chains (right panel) of the model parameters for Participant 1 in the Bissett and Logan (2011) data set obtained with the individual BPA**.

In this section, we present the results of fitting the data of Participant 1 with the individual BPA. See the examples folder for the data set. Using three CPU cores, the sampling took approximately 23 min with BEESTS. The same analysis took about 15 h with WinBUGS. The top left panel of Figure 6 shows the required data format for the individual analysis. Figure 7 shows the posterior and prior distributions (left panel; option Posterior distributions) and the MCMC chains (right panel; option MCMC chains) for the six model parameters. The prior distributions are adequately updated; the posteriors are substantially narrower than the priors. The posterior distributions and the three MCMC chains do not show signs of convergence problems. All $\hat{R}$ values were lower than 1.05. The middle left panel of Figure 6 shows the summary statistics of the posterior distribution of the model parameters (option Summary statistic). The posterior distributions are estimated well as evidenced by the relatively small posterior standard deviations. The go parameters are generally estimated more precisely than the stop parameters because the go parameters are estimated based on the go RTs as well as the signal-respond RTs and are therefore better constrained by the data.

**Figure 8:**
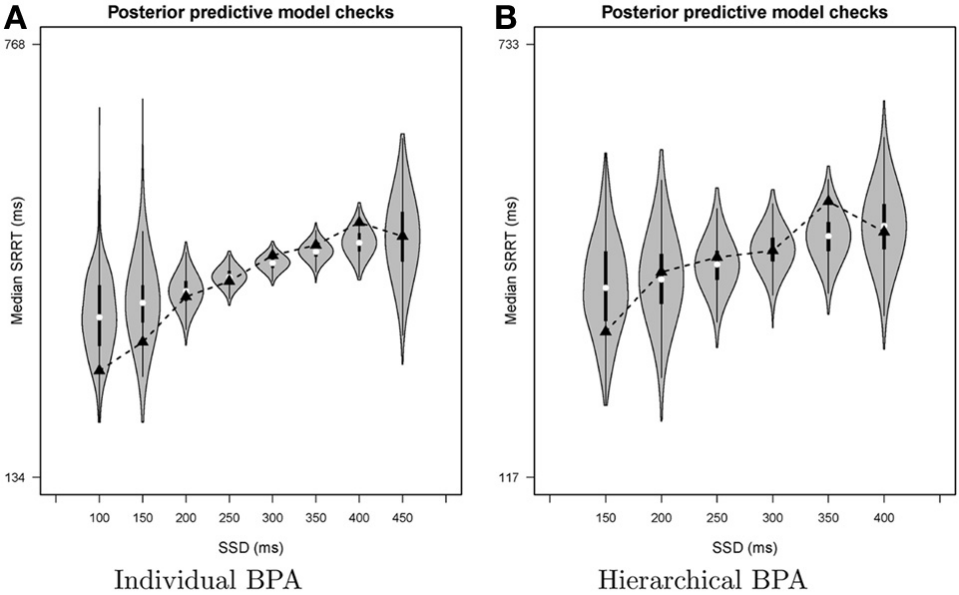
**Results of the posterior predictive model checks for Participant 1 in the Bissett and Logan (2011) data set with the individual BPA (panel A) and the hierarchical BPA (panel B)**. See text for a detailed description of the posterior predictive analyses. For each SSD, the figures show the observed median signal-respond RT (black triangle), a density plot of the predicted median signal-respond RTs (gray violin plot), a boxplot ranging from the 25th to the 75th percentile of the predicted median signal-respond RTs, and the median of the predicted median signal-respond RTs (white circle). SRRT = signal-respond RT.

The bottom left panel of Figure 6 shows the summary of the posterior predictive model checks (option Goodness-of-fit) using 1000 samples from the joint posterior of the model parameters (Samples = 1000). As mentioned above, we advise users to assess model fit only on SSDs with a reasonable number of observed signal-respond RTs. For instance, we assessed goodness-of-fit only on SSDs with at least 10 observed signal-respond RTs. The one-sided *p* values on these five SSDs (i.e., 200, 250, 300, 350, and 400 ms) are far from 0 or 1 and the two-sided *p* values are all above 0.05. The left panel of Figure 8 shows the corresponding graphical summary for the model checks. For the selected SSDs, the observed median signal-respond RTs (i.e., black triangles) are well within the 2.5th and 97.5th percentile of the predicted median signal-respond RTs (see gray violin plots), and are adequately approximated by the median of the predicted median signal-respond RTs (i.e., white circles). The results of the posterior predictive model checks indicated thus that the BEESTS analysis appropriately accounted for the observed data.

### 7.2. Hierarchical analysis

As explained above, the hierarchical approach has the potential to provide accurate parameter estimates with relatively few observations per participant. To illustrate the benefits of the hierarchical approach over the individual BPA with scarce data, this section presents the results of fitting a subsample of the observations from the Bissett and Logan (2011) data set with the hierarchical as well as the individual BPA. For each of the 20 participants, we fit a randomly selected 90 go RTs, 30 signal-respond RTs, and 30 successful inhibitions with the hierarchical BPA. We then compared the results from the hierarchical analysis to the results from fitting the same subsample of data with the individual BPA. Using three CPU cores, the hierarchical analysis took approximately 3.5 h with BEESTS. The same analysis took about 100 h with WinBUGS.

**Figure 9:**
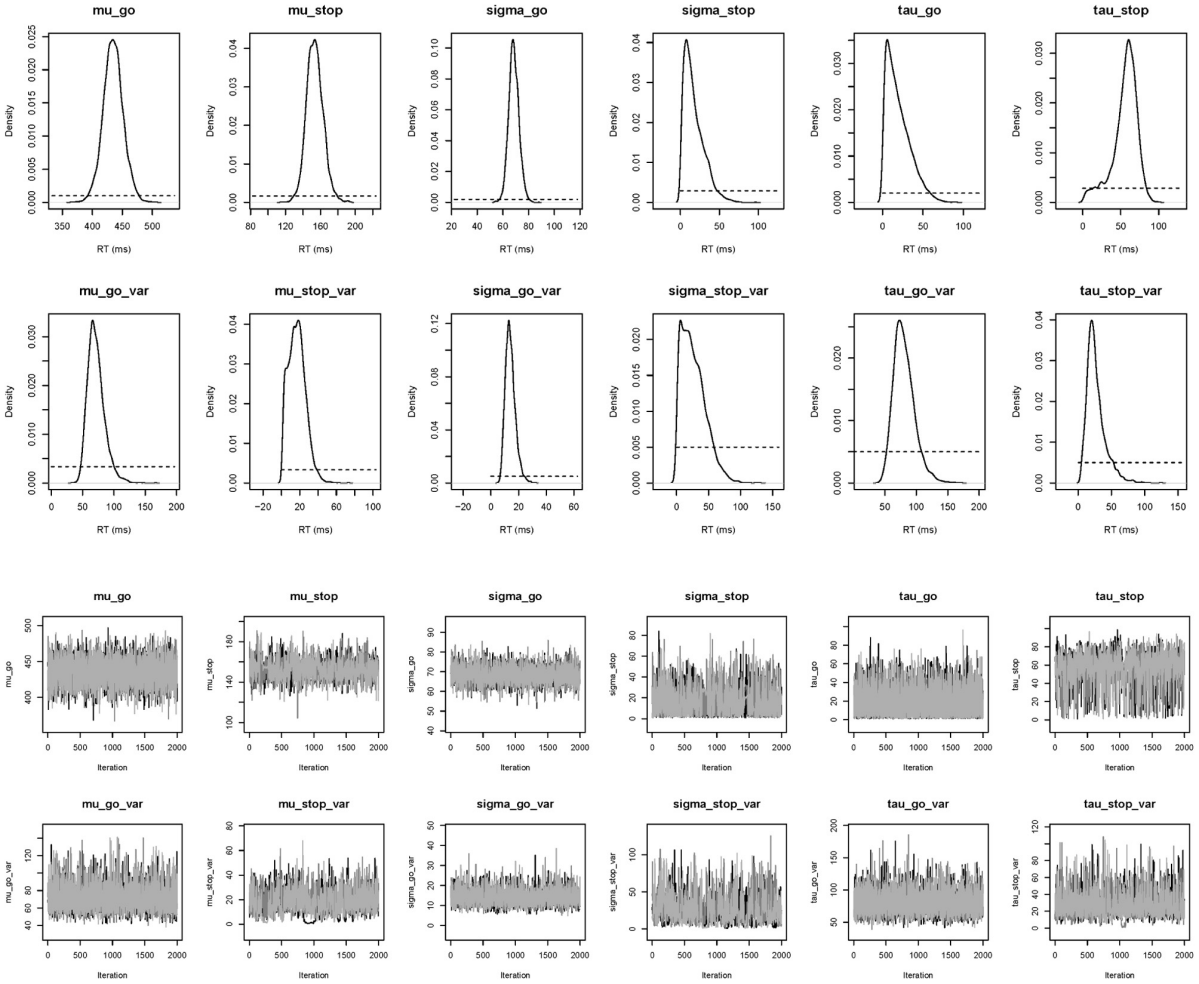
**Posterior distributions and MCMC chains of the group-level model parameters in the Bissett and Logan (2011) data set obtained with the hierarchical BPA**. The first and third rows show posterior (black solid line) and prior distributions (black dotted line) and MCMC trace plots for the group-level mean parameters, respectively. The second and fourth rows show posterior and prior distributions and trace plots for the group-level variability (i.e., group-level standard deviation) parameters, respectively.

The top right panel of Figure 6 shows the required data format for the hierarchical analysis. Figure 9 shows the posterior and prior distributions (top panel) and the MCMC chains (bottom panel) for the group-level mean and standard deviation parameters. The prior distribution of the group-level parameters are adequately updated; the posteriors are substantially narrower than the priors and the chains have mixed well. The $\hat{R}$ values for all group-level and individual parameters were lower than 1.05. The middle right panel of Figure 6 shows the summary statistics of the posterior distribution of the group-level mean and standard deviation parameters. The posterior distributions are estimated relatively precisely. Note that if the Estimates for All option is selected, BEESTS also produces output (i.e., density plots of the posteriors, MCMC trace plots, and summary statistics) for the individual go and stop parameters for each participant separately.

The bottom right panel of Figure 6 shows the summary of the posterior predictive model checks for Participant 1 using 1000 samples from the joint posterior of the participant-level model parameters obtained with the hierarchical BPA. All posterior predictive *p* values are well within an acceptable range. Note, however, that the median signal-respond RTs—observed and predicted—are based on only a few observations. The right panel of Figure 8 shows the corresponding graphical summary of the posterior predictive model checks. All observed median signal-respond RTs are well within the range of the median signal-respond RTs predicted by the joint posterior of the model parameters. Due to the scarcity of the data, however, there is large uncertainty in the predicted median signal-respond RTs. Compare the results of the posterior predictive model checks in the two panels of Figure 8. The violin plots in the left panel show the predicted median signal-respond RTs from the individual analysis of the data of Participant 1 based on the full 1200 trials. The violin plots in the right panel show the predicted median signal-respond RTs from the hierarchical analysis of the data of Participant 1 based on a subsample of only 150 trials. Because the hierarchical analysis is based on substantially fewer observations than the individual analysis of the full data set presented in the previous section, the predicted median signal-respond RTs in the right panel are more spread out than the predicted median signal-respond RTs in the left panel. Posterior predictive *p* values resulting from such unstable observed and predicted median signal-respond RTs should be interpreted with caution.

**Figure 10:**
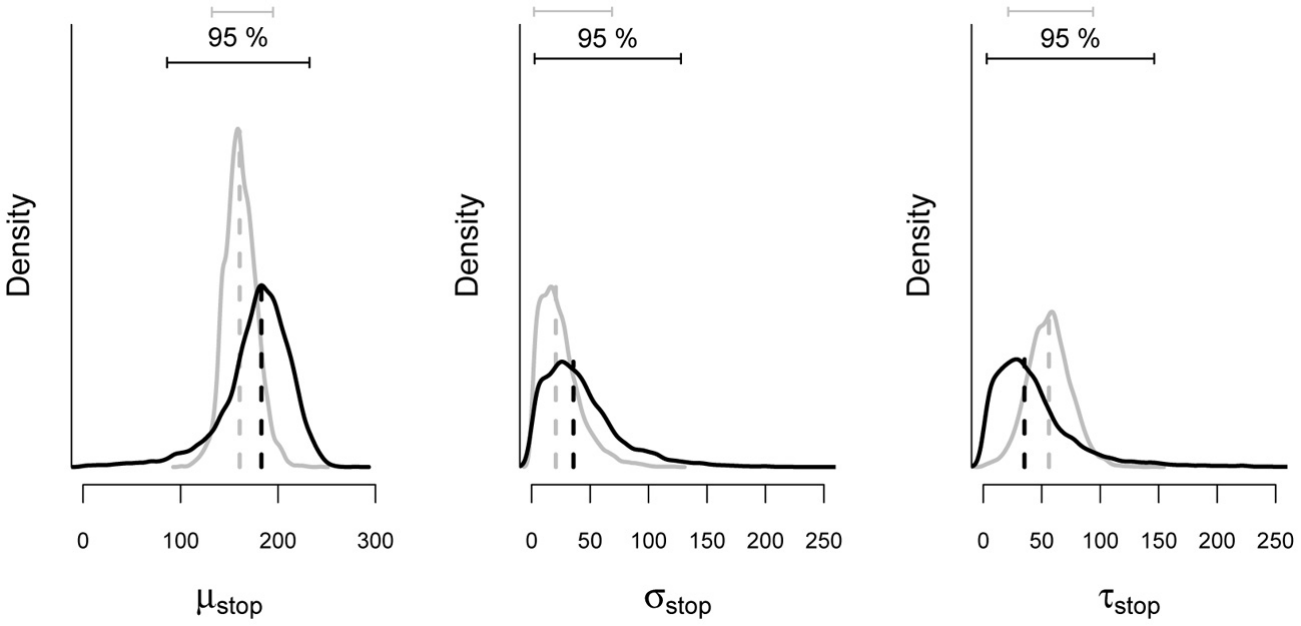
**Posterior distribution of the stop parameters estimated from a subsample of the data of Participant 1 with the individual and the hierarchical BPA**. The solid black and gray lines show the posterior distribution of the stop parameters and the corresponding 95% Bayesian credible intervals obtained with the individual and the hierarchical BPA, respectively. The dashed black and gray lines show the median of the posterior distributions obtained with the individual and the hierarchical BPA, respectively (see also Matzke et al., 2013).

To illustrate the benefits of the hierarchical approach over the individual BPA with scarce data, we compared the parameter estimates from the hierarchical analysis with estimates obtained from the individual analysis of the same subsample of 150 trials. As mentioned above, hierarchical modeling generally results in more accurate and less variable estimates than single-level estimation. Figure 10 shows the posterior distribution of the stop parameters of Participant 1 obtained with the hierarchical and the individual BPA using the same subsample of 150 observations. The gray density plots show the posterior distribution of the stop parameters from the hierarchical BPA. The black density plots show the posterior distribution of the stop parameters from the individual analysis. The posterior distributions of the stop parameters estimated with the hierarchical approach are less variable (i.e., smaller 95% Bayesian credible interval) than the posteriors estimated with the individual BPA. Also, the posterior medians from the hierarchical analysis are—as expected—shrunk toward their corresponding group mean (see also Matzke et al., 2013).

## 8. Discussion

The horse-race model presents various opportunities to estimate the latency of response inhibition in the stop-signal paradigm. Most methods, however, only focus on deriving a summary measure of SSRT. Recently, Matzke et al. (2013) have developed the BPA that allows for the estimation of the entire distribution of stopping latencies. The goal of the present paper was to promote the widespread application of the Bayesian analysis of stop-signal data by introducing BEESTS, a relatively fast and user-friendly software implementation of the BPA. BEESTS provides users with a range of output options, such as summary statistics of the posterior distribution of the parameters and various diagnostic tools to assess the quality of the estimates. Importantly, BEESTS is equipped with an easy-to-use graphical user interface.

BEESTS can be applied to individual as well as hierarchical stop-signal data. The advantage of the individual approach lies in its simplicity. The advantage of the hierarchical approach lies in its potential to provide accurate parameter estimates with relatively few observations per participant. The choice between the individual and the hierarchical approach in practical applications depends on a delicate balance between the quality of the data, the number of participants, the number of trials per participant, and whether users are interested in obtaining accurate parameter estimates on the participant level in order to examine individual differences or focus on group comparisons and are satisfied with interpreting only the group-level parameters. Prior to data collection, users are encouraged to generate synthetic data with varying number of trials and participants, fit the data in BEESTS, and inspect the parameter estimates in order to assess the expected uncertainty of the model parameters under the different scenarios and modeling approaches.

BEESTS assumes that go RTs and SSRTs are ex-Gaussian distributed and relies on Bayesian parameter estimation to obtain estimates for the go and stop parameters. Note, however, that the BPA itself does not hinge on the particular parametric form used to summarize the distributions, nor is it heavily influenced by the exact choice of the prior distributions. In our experience, the ex-Gaussian assumption and the corresponding (group-level and hyper) prior distributions implemented in BEESTS provide a reasonable default setting. Nevertheless, interested users may adapt the source code (https://github.com/twiecki/stopsignal) to accommodate alternative parametric assumptions or different prior settings. Also, the posterior predictive model check implemented in BEESTS using the median signal-respond RT is only one of many possible approaches to assess the goodness-of-fit of the model. Users may adapt the source code to implement posterior predictive model checks using alternative test statistics (see Matzke et al., 2013).

## 9. Conclusion

Here we introduced a user-friendly software package—BEESTS—that allows for the efficient estimation of entire SSRT distributions using MCMC sampling. BEESTS allows researchers to rigorously address important questions about the variability of stopping latencies, such as the relationship between mean SSRT and SSRT variance. Similarly, BEESTS enables investigators to assess differences in the shape of go RT and SSRT distributions between clinical populations or experimental groups. BEESTS therefore facilitates the interpretation of stop-signal data and may open fruitful new avenues for response inhibition research.

### Conflict of interest statement

The authors declare that the research was conducted in the absence of any commercial or financial relationships that could be construed as a potential conflict of interest.

## A. Appendix

### A.1 Prior distribution of the model parameters

This appendix presents the prior distributions of the model parameters in the BEESTS implementation of the Bayesian parametric approach (BPA). The name of each parameter as shown in the BEESTS output is in brackets.

### A.1.1 Individual BPA

The priors for the go and stop parameters are uniform distributions, spanning a plausible but wide range of values. BEESTS relies on slightly more diffuse priors than the WinBUGS implementation of the BPA (see Matzke et al., 2013):

(A1) $$\begin{array}{l} \mu_{\text{go}}(\text{mu}\_\text{go})\sim \text{Uniform}(0.001,1000)\\ \sigma_{\text{go}}(\text{sigma}\_\text{go})\sim \text{Uniform}(1,500)\\ \tau_{\text{go}}(\text{tau}\_\text{go})\sim \text{Uniform}(1,500)\\ \mu_{\text{stop}}(\text{mu}\_\text{stop})\sim \text{Uniform}(0.001,600)\\ \sigma_{\text{stop}}(\text{sigma}\_\text{stop})\sim \text{Uniform}(1,350)\\ \tau_{\text{stop}}(\text{tau}\_\text{stop})\sim \text{Uniform}(1,350). \end{array}$$

### A.1.2 Hierarchical BPA

***A.1.2.1 Individual parameters.*** The hierarchical BPA assumes that the μ_go_, σ_go_, τ_go_, μ_stop_, σ_stop_, and τ_stop_ parameters of each participant *j* = 1, …, *J* come from truncated normal group-level distributions. The group-level distributions are themselves characterized by a group mean (μ) and a group standard deviation (σ) parameter. The WinBUGS implementation relies on normal group-level distributions that are truncated only at the lower end, whereas BEESTS uses normal distributions that are truncated at the lower *and* the upper ends:

(A2) $$\begin{array}{l} \mu_{{\text{go}}_{j}}(\text{mu}\_\text{go}.\text{subj})\sim \text{Normal}(\mu_{\mu_{\text{go}}},\sigma_{\mu_{\text{go}}})[0.001,1000]\\ \sigma_{{\text{go}}_{j}}(\text{sigma}\_\text{go}.\text{subj})\sim \text{Normal}(\mu_{\sigma_{\text{go}}},\sigma_{\sigma_{\text{go}}})[1,500]\\ \tau_{{\text{go}}_{j}}(\text{tau}\_\text{go}.\text{subj})\sim \text{Normal}(\mu_{\tau_{\text{go}}},\sigma_{\tau_{\text{go}}})[1,500]\\ \mu_{{\text{stop}}_{j}}(\text{mu}\_\text{stop}.\text{subj})\sim \text{Normal}(\mu_{\mu_{\text{stop}}},\sigma_{\mu_{\text{stop}}})[0.001,600]\\ \sigma_{{\text{stop}}_{j}}(\text{sigma}\_\text{stop}.\text{subj})\sim \text{Normal}(\mu_{\sigma_{\text{stop}}},\sigma_{\sigma_{\text{stop}}})[1,350]\\ \tau_{{\text{stop}}_{j}}(\text{tau}\_\text{stop}.\text{subj})\sim \text{Normal}(\mu_{\tau_{\text{stop}}},\sigma_{\tau_{\text{stop}}})[1,350]. \end{array}$$

***A.1.2.2 Group-level parameters.*** The priors for the group mean and group standard deviations are uniform distributions. Note that the WinBUGS implementation uses censored normal priors for the group-level means and relies on slightly less diffuse priors for the group-level standard deviations than BEESTS:

(A3) $$\begin{array}{l} \mu_{\mu_{\text{go}}}(\text{mu}\_\text{go})\sim \text{Uniform}(\text{0}.\text{001},\text{1000})\\ \sigma_{\mu_{\text{go}}}(\text{mu}\_\text{go}\_\text{var})\sim \text{Uniform}(0.01,300)\\ \mu_{\sigma_{\text{go}}}(\text{sigma}\_\text{go})\sim \text{Uniform}(1,500)\\ \sigma_{\sigma_{\text{go}}}(\text{sigma}\_\text{go}\_\text{var})\sim \text{Uniform}(0.01,200)\\ \mu_{\tau_{\text{go}}}(\text{tau}\_\text{go})\sim \text{Uniform}(1,500)\\ \sigma_{\tau_{\text{go}}}(\text{tau}\_\text{go}\_\text{var})\sim \text{Uniform}(0.01,200)\\ \mu_{\mu_{\text{stop}}}(\text{mu}\_\text{stop})\sim \text{Uniform}(0.001,600)\\ \sigma_{\mu_{\text{stop}}}(\text{mu}\_\text{stop}\_\text{var})\sim \text{Uniform}(0.01,300)\\ \mu_{\sigma_{\text{stop}}}(\text{sigma}\_\text{stop})\sim \text{Uniform}(1,350)\\ \sigma_{\sigma_{\text{stop}}}(\text{sigma}\_\text{stop}\_\text{var})\sim \text{Uniform}(0.01,200)\\ \mu_{\tau_{\text{stop}}}(\text{tau}\_\text{stop})\sim \text{Uniform}(1,350)\\ \sigma_{\tau_{\text{stop}}}(\text{tau}\_\text{stop}\_\text{var})\sim \text{Uniform}(0.01,200). \end{array}$$
